# Hub metastatic gene signature and risk score of breast cancer patients with small tumor sizes using WGCNA

**DOI:** 10.1007/s12282-024-01627-w

**Published:** 2024-08-27

**Authors:** Yu-Tien Chang, Zhi-Jie Hong, Hsueh-Han Tsai, An-Chieh Feng, Tzu-Ya Huang, Jyh-Cherng Yu, Kuo-Feng Hsu, Chi-Cheng Huang, Wei-Zhi Lin, Chi-Ming Chu, Chia-Ming Liang, Guo-Shiou Liao

**Affiliations:** 1 School of Public Health, National Defense Medical Center, Taipei City, Taiwan; 2 Division of General Surgery, Department of Surgery, Tri-Service General Hospital, National Defense Medical Center, No. 325, Sec. 2, Chenggong Rd., Neihu Dist., Taipei City, 114202 Taiwan; 3 Department of Surgery, Taipei Veterans General Hospital, No.201, Sec. 2, Shipai Rd., Beitou District, Taipei City, 11217 Taiwan; 4 AIoT Center, Tri-Service General Hospital, Taipei City, Taiwan; 5 Comprehensive Breast Health Center, Taipei Veterans General Hospital, No. 201, Sec. 2, Shipai Rd., Beitou District, Taipei City, 11217 Taiwan; 6 Institute of Epidemiology and Preventive Medicine, College of Public Health, National Taiwan University, No. 17, Xuzhou Rd., Taipei City, 100 Taiwan

**Keywords:** Breast cancer, Small tumor size, LASSO cox regression, Weighted gene co-expression network analysis, Distant metastasis-free survival

## Abstract

**Background:**

Breast cancer (BC) is the most common cancer in women and accounts for approximately 15% of all cancer deaths among women globally. The underlying mechanism of BC patients with small tumor size and developing distant metastasis (DM) remains elusive in clinical practices.

**Methods:**

We integrated the gene expression of BCs from ten RNAseq datasets from Gene Expression Omnibus (GEO) database to create a genetic prediction model for distant metastasis-free survival (DMFS) in BC patients with small tumor sizes (≤ 2 cm) using weighted gene co-expression network (WGCNA) analysis and LASSO cox regression.

**Results:**

ABHD11, DDX39A, G3BP2, GOLM1, IL1R1, MMP11, PIK3R1, SNRPB2, and VAV3 were hub metastatic genes identified by WGCNA and used to create a risk score using multivariable Cox regression. At the cut-point value of the median risk score, the high-risk score (≥ median risk score) group had a higher risk of DM than the low-risk score group in the training cohort [hazard ratio (HR) 4.51, *p* < 0.0001] and in the validation cohort (HR 5.48, *p* = 0.003). The nomogram prediction model of 3-, 5-, and 7-year DMFS shows good prediction results with *C*-indices of 0.72–0.76. The enriched pathways were immune regulation and cell–cell signaling. EGFR serves as the hub gene for the protein–protein interaction network of PIK3R1, IL1R1, MMP11, GOLM1, and VAV3.

**Conclusion:**

Prognostic gene signature was predictive of DMFS for BCs with small tumor sizes. The protein–protein interaction network of PIK3R1, IL1R1, MMP11, GOLM1, and VAV3 connected by EGFR merits further experiments for elucidating the underlying mechanisms.

**Graphical abstract:**

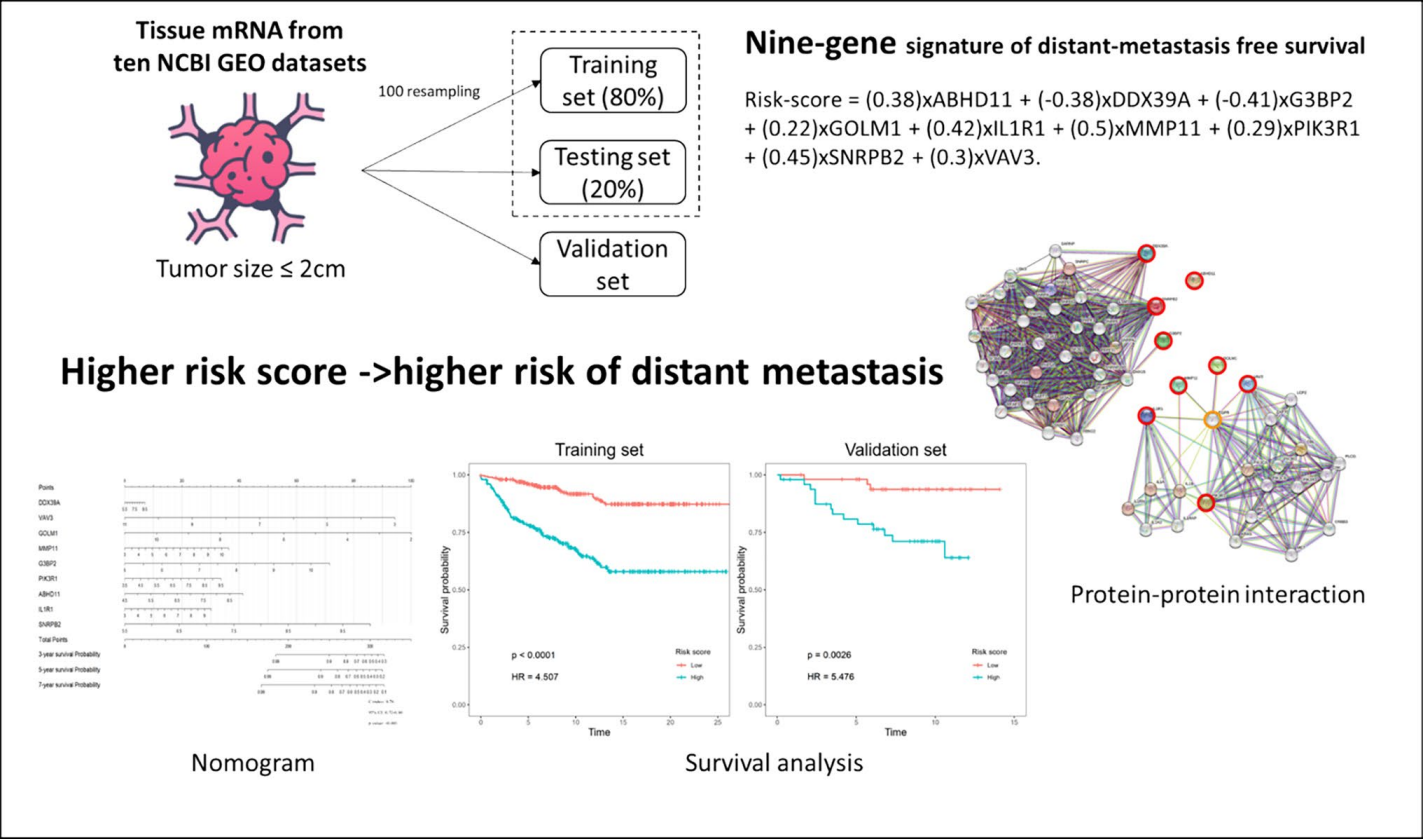

**Supplementary Information:**

The online version contains supplementary material available at 10.1007/s12282-024-01627-w.

## Introduction

Breast cancer is still the most common cancer in women. In 2020, 2.3 million women were diagnosed with breast cancer, with 685,000 reported deaths globally [1]. The heterogeneity of breast cancer is attributed to differences in the genomic, epigenetic, transcriptomic, and proteomic characteristics of the cancer cells. These factors have an impact on tumor properties such as proliferation, apoptosis, metastasis, and therapeutic response [2]. In clinical diagnosis, the histological status of biomarkers, such as estrogen receptor (ER), progesterone receptor (PR), and Human Epidermal Growth factor Receptor-2 (HER2), and Ki67, are critical characteristics for prognosis estimation. The two most important factors are tumor size and lymph-node metastasis status. As for the disease development and progression, larger tumors and positive lymph-node metastases are often associated with poor outcomes in breast cancer. However, it has been found that some patients with small tumors have severe lymph-node metastasis, resulting in poor prognosis [3, 4]. Moreover, in extensive node-positive breast cancers, very small tumor size may be a surrogate for biologically aggressive disease [5]. In the same case of lymph-node metastasis, patients with small tumors have a higher breast cancer-specific mortality rate [6]. However, the underlying mechanism remains elusive.

Since the status of the biomarkers is essential to prognosis estimation, and tumor size and lymph-node distant metastases are the two factors for survival, it is critical to identify the missing link first by analyzing the genomic characteristics. However, the regulatory relationships between genes are of high complexity. Moreover, each regulatory network has a group of highly related genes called the Hub gene. Therefore, we employed Weighted Gene Co-expression Network Analysis (WGCNA), a bioinformatics approach. By using WGCNA, we can simplify up to thousands of genes into several highly correlated gene modules, construct a free-scale network through weighting and co-expression, and explore the module structure, gene and module (module membership information), module and module information in the network, as long as modules (eigengene network methodology) and their association with clinical features [6].

In this study, we aim to identify candidate genes affecting distant metastasis in breast cancer patients with small-size tumors through WGCNA and LASSO Cox-hazards model to establish a genetic risk score for identifying high-risk patients and improve treatment strategies to prevent tumor progression.

## Materials and methods

### Immunohistochemical subtyping

We analyzed the number of positively stained cell using immunohistochemistry (IHC). A sample was considered positive if the percentage of ER or PR was greater than 1%. The criteria for defining HER2 positivity were IHC 3 + or fluorescence in situ hybridization (FISH) positivity.

### RNAseq data

We integrated nine mRNA datasets of breast cancers as a training cohort, GSE6532, GSE6532, GSE9195, GSE11121, GSE16446, GSE25066, GSE45255, GSE58984, and GSE158309 (Table S1) downloaded from the National Center for Biotechnology Information (NCBI) Gene Expression Omnibus (GEO) database (https://www.ncbi.nlm.nih.gov/geo). There are 2013 patients in total. The selection criteria include primary breast tumors from human tissues, clinical characteristics of females, distant metastases-free survival (DMFS), tumor size, lymph-node status, and at least one clinical feature of grading, ER, PR, and HER2. After filtering out tumor size > 2 cm and missing data of distant metastasis status and time, 598 patients were included in the prognostic biomarker analysis that contained 474 patients without distant metastases and 124 patients with distant metastases. All analyses in this study were performed in the R software (version 4.2.2; https://www.r-project.org/). Training cohorts integrated from nine GSE datasets were normalized to remove batch effects using the linear models for microarray RNA-seq data (LIMMA) R package and “removeBatchEffect” function. GSE20685, which contained 17 patients with distant metastasis and 84 patients without distant metastasis, was used to validate the prediction model.

### Prognostic prediction model

**Fig. 1:**
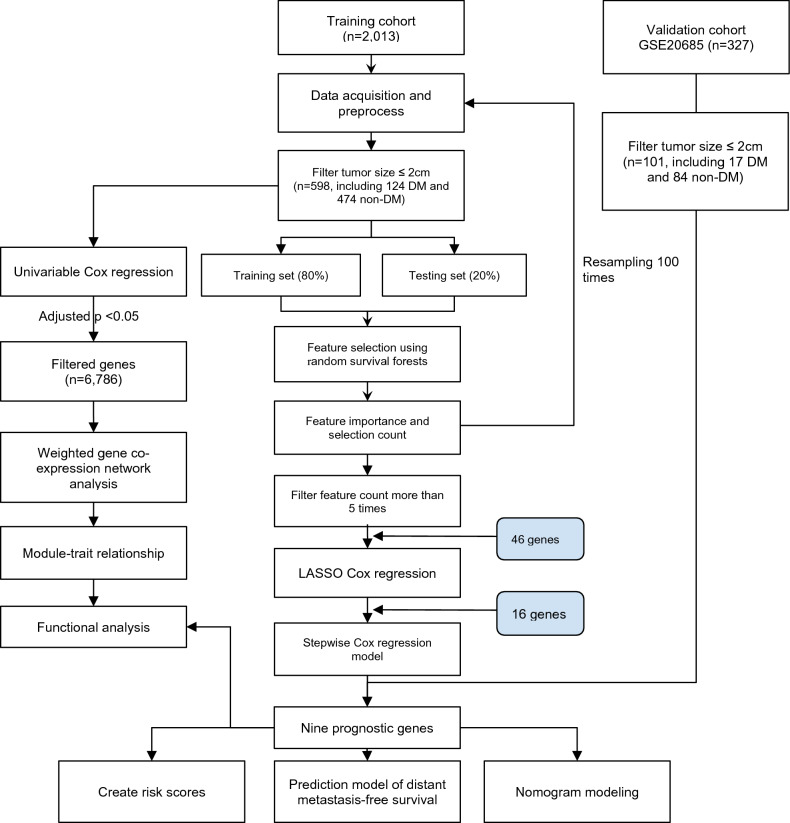
The study workflow

**Fig. 2:**
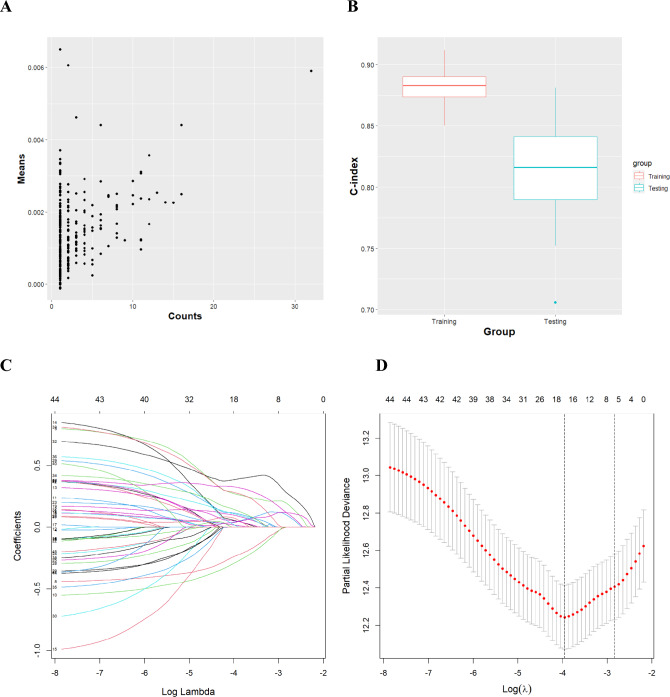
The process of selecting the optimal prognostic gene combination using random survival forests (**A**, **B**) and Lasso Cox regression (**C**, **D**). **A** The scatter plot of counts of genes being selected (*x*-axis) and average feature importance (y-axis); **B** box plots of average *C*-indices of training sets and testing sets in 100 loops of random forest survival analysis. 46 genes were filtered by the number of being selected ≥ 5 in 100-round samplings and then entered into the Lasso Cox regression for obtaining the optimal gene combination. **C** *λ* selection diagram. **D** The two dotted lines indicated two particular values of *λ*. The left side was *λ*min and the right side was *λ*1se. The *λ*min was selected to build the model for accuracy in our study

As shown in the study workflow (Fig. 1), we first resampled 80% data into the training set and 20% data into the testing set 100 times. Next, we used random survival forests (rsf) to identify important genes for distant metastasis. Then, we summarized the number of selected genes, and calculated the variable importance for corresponding genes. We plotted the scatter plot of gene count and variable importance in 100 times analysis (Fig. 2A). There was a positive linear relationship between gene count and variable importance if the gene count was over five. Namely, genes selected 5 times in 100 resampling results were more likely to have higher importance for distant metastasis prediction.

The concordance index or C-index is a generalization of the area under the ROC curve (AUC) [7] that is used to evaluate predictions made by a model. In 100-round analyses, the C-indices of training and testing sets were 0.88 ± 0.01 and 0.81 ± 0.03, respectively (Fig. 2B). The average prediction efficacy was good, which indicated that selected significant genes were the potential biomarkers.

The LASSO Cox regression was performed using the “glmnet” R package to obtain the optimal gene combination. The degree of Lasso regression complexity was controlled by the appropriate parameter *λ*, and *λ* was selected to build the model for accuracy. The *λ* selection diagram is shown in Fig. 2C, D. The model constructed by *λ*1se was the simplest. It used a small number of genes, while *λ*_min_ had a higher accuracy rate and used a larger number of genes. The *λ*_min_ was selected to build the model for accuracy in our study.

Finally, the prognostic risk score was built using Cox regression according to the expression levels of prognostic RNAs and their prognostic coefficients with the calculation formula as follows:

### Cell line expression data

Gene expression data of prognostic genes in various breast cancer cell lines (*n* = 60) were derived from Cancer Cell Line Encyclopedia (CCLE) and Dependency Map (DepMap) 22Q2. They were used to plot the heatmap using the R package “pheatmap” and investigate the prognostic gene expression in various characteristic BC cell lines.

### Weighted gene co-expression network analysis

We filter out genes whose adjusted *p* values (false discovery rate) < 0.05 in the univariable Cox regression. 6786 genes were left to construct the co-expression network and computed module–trait relationship using weighted gene co-expression network analysis (WGCNA). First, we used adjacency and soft threshold power of *β* to calculate co-expression similarity. Second, we used the TOMsimilarity function to convert a topological overlap matrix to a distance matrix and build hierarchical clustering to identify modules. Then, cutting clusters were employed by the “cutreeDynamic” function. After merging-related modules with the “mergeCloseModules” function, we recalculated the module eigengenes to their corresponding modules and calculated the module–trait correlation to identify significant clinical modules. Finally, functional annotation analysis of the modules was performed using the "userListEnchment" function of the WGCNA package.

### Nomogram and protein–protein interaction networks

A nomogram was constructed using the “rms” package to visualize the prediction value of prognostic genes further. The *C*-index was calculated to evaluate the discriminative ability of the nomogram, and calibration curves were drawn to show the consistency between the predicted 3-year, 5-year, and 7-year endpoint events and the authentic outcomes.

The STRING database collects, scores, and integrates information on protein–protein interactions from various public databases. For the functional annotation of biomarkers, the prognostic genes were adopted to conduct protein–protein interaction networks using STRING (https://string-db.org/).

## Results

### Patients and demographic

We summarized essential characteristics of breast cancer patients with tumor size ≤ 2 cm in the training cohort by distant metastasis-free survival (DMFS) time, age, tumor grade, lymph node status, biomarker status (ER, PR, and HER2), and the Prediction Analysis of Microarray 50 (PAM50) results (Table 1). There were 474 patients without distant metastasis and 124 patients with distant metastasis. All patients were female and the average age was 58 years old. The average follow-up time is 10 years. The ER, PR, and PAM50 subtyping were significantly associated with DMFS, while others were not significantly associated.

**Table 1:** The characteristics of breast cancer patients with tumor size ≤ 2 cm

	Distant metastasis-free survival event		Total (*N* = 598)	*p* value
	No (*N* = 474)	Yes (*N* = 124)		
Time (years)				
Mean (SD)	11.4 (6.30)	4.88 (3.89)	10.0 (6.44)	< 0.001
Median [min, max]	9.94 [0, 26.3]	3.58 [0, 13.4]	8.45 [0, 26.3]	
Age				
Mean (SD)	58.5 (12.1)	57.3 (12.6)	58.3 (12.2)	0.637
Median [min, max]	58.5 [32.0, 89.0]	58.0 [30.0, 85.0]	58.0 [30.0, 89.0]	
Grading				
I	118 (24.9%)	19 (15.3%)	137 (22.9%)	0.181
II	272 (57.4%)	75 (60.5%)	347 (58.0%)	
III	84 (17.7%)	30 (24.2%)	114 (19.1%)	
Lymph-node status				
−	376 (79.3%)	91 (73.4%)	467 (78.1%)	0.363
+	98 (20.7%)	33 (26.6%)	131 (21.9%)	
ER				
−	48 (10.1%)	40 (32.3%)	88 (14.7%)	< 0.001
+	426 (89.9%)	84 (67.7%)	510 (85.3%)	
PR				
−	118 (24.9%)	59 (47.6%)	177 (29.6%)	< 0.001
+	356 (75.1%)	65 (52.4%)	421 (70.4%)	
HER2				
−	38 (8.0%)	8 (6.5%)	46 (7.7%)	1^$^
+	12 (2.5%)	2 (1.6%)	14 (2.3%)	
PAM50				
Luminal A	43 (9.1%)	22 (17.7%)	65 (10.9%)	< 0.001
Luminal B	33 (7.0%)	20 (16.1%)	53 (8.9%)	
Her2-enriched	217 (45.8%)	31 (25.0%)	248 (41.5%)	
Basal-like	136 (28.7%)	37 (29.8%)	173 (28.9%)	
Normal-like	45 (9.5%)	14 (11.3%)	59 (9.9%)	

The *p* values were derived from Pearson correlation and Student’s *t* test

^$^Fisher’s exact test

### Construction and validation of the prognostic signature

After the feature selection using rsf, 46 genes were selected to enter the LASSO Cox regression model to reduce the dimension of genes. Then, 16 genes were selected and entered into the stepwise Cox regression model. Finally, an optimal gene set of nine genes, Abhydrolase domain-containing protein 11 (ABHD11), DExD-box helicase 39A (DDX39A), G3BP Stress Granule Assembly Factor 2 (G3BP2), Golgi membrane protein 1 (GOLM1), Interleukin 1 Receptor Type 1 (IL1R1), Ｍatrix metallopeptidase 11 (MMP11), Phosphoinositide-3-Kinase Regulatory Subunit 1 (PIK3R1), small nuclear ribonucleoprotein polypeptide B2 (SNRPB2), and vav guanine nucleotide exchange factor 3 (VAV3) were selected to build the prediction model using multivariable Cox regression. GOLM1 and VAV3 were suppressor genes of distant metastasis, while the others were oncogenes (Table 2).

**Table 2:** Prognostic genes for predicting the distant metastasis recurrence

Gene	Gene name	logFC	Univariable Cox regression			Multivariable Cox regression		
			HR	*p* value	FDR	Coef	HR	*p* value
ABHD11	Abhydrolase domain containing 11	0.288	1.987	< 0.001	< 0.001	0.383	1.987	0.136
DDX39A	Dexd-box helicase 39A	0.185	2.24	< 0.001	< 0.001	− 0.375	2.24	0.001**
G3BP2	G3Bp stress granule assembly factor 2	0.226	2.033	< 0.001	< 0.001	− 0.41	2.033	0.001**
GOLM1	Golgi membrane protein 1	− 0.079	0.76	0.002	0.008	0.222	0.76	0.037*
IL1R1	Interleukin 1 receptor type 1	0.265	1.307	0.01	0.023	0.423	1.307	0.148
MMP11	Matrix metallopeptidase 11	0.384	1.308	0.001	0.004	0.497	1.308	0.026*
PIK3R1	Phosphoinositide-3-kinase regulatory subunit 1	0.26	1.342	0.006	0.016	0.292	1.342	0.031*
SNRPB2	Small nuclear ribonucleoprotein polypeptide B2	0.268	2.788	< 0.001	< 0.001	0.449	2.788	0.017*
VAV3	Vav guanine nucleotide exchange factor 3	− 0.29	0.732	< 0.001	0.001	0.296	0.732	0.032*

*logFC* log2 fold change of gene expression distant metastasis versus non-distant metastasis, *Coef* coefficient, *HR* hazard ratio, *FDR* false discovery rate

**Fig. 3:**
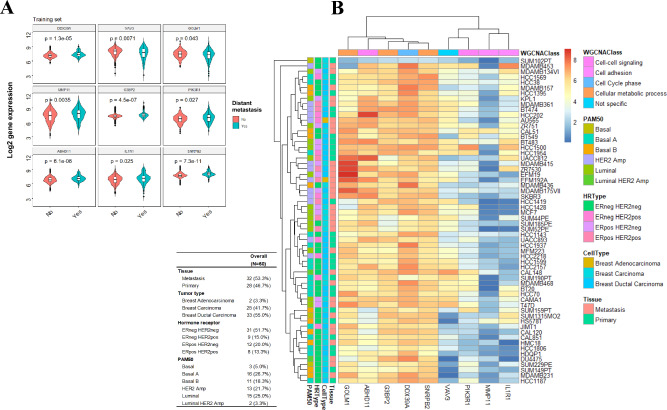
The gene expression of prognostic genes. **A** The violin plots of gene expression between distant metastasis (DM) group and non-DM group using the training cohort. **B** The heatmap of prognostic gene expression using Cancer Cell Line Encyclopedia and Dependency Map data

Gene expression of nine prognostic genes in tissue samples and various BC cell lines is shown in Fig. 3. All prognostic genes are differentially expressed between primary tissues with distant metastasis (DM) and non-DM (Fig. 3A). In the gene expression heatmap of 60 BC cell lines, prognostic genes were cluster into two gene sets, geneset 1 of GOLM1, ABHD11, G3BP2, DDX39A, SNRPB2, as well as geneset 2 of VAV3, PIK3R1, MMP1, and IL1R1. In general, geneset 1 expresses a relatively higher expression than geneset2 across all cells (Fig. 3B).

We performed univariable- and multivariable Cox regression analyses using the training cohort to determine whether nine prognostic genes can serve as an independent prognostic factor. As revealed by the multivariable Cox regression analysis, except for ABHD11, the other genes DDX39A, G3BP2, GOLM1, IL1R1, MMP11, PIK3R1, SNRPB2, and VAV3 were independent prognostic factors (Table 2). We still retained ABHD11 in the prediction model because of better overall prediction efficacy. Based on the coefficients of the Cox regression model and gene expression values of the nine genes, the risk score for each patient was calculated as follows: risk‑score = (0.38) × ABHD11 + (− 0.38) × DDX39A + (− 0.41) × G3BP2 + (0.22) × GOLM1 + (0.42) × IL1R1 + (0.5) × MMP11 + (0.29) × PIK3R1 + (0.45) × SNRPB2 + (0.3) × VAV3.

At the cut-point value of the median risk score, all patients in integrated data were classified into a high-risk group (*n* = 299; ≥ median risk score) and a low‑risk group (*n* = 299; < median risk score).

**Fig. 4:**
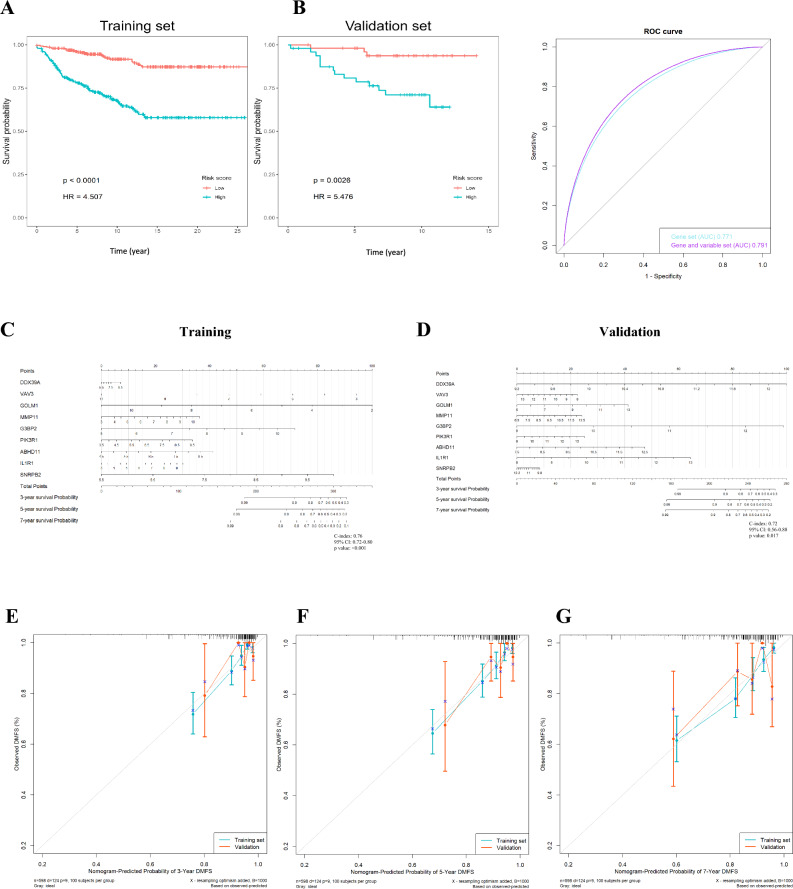
Prediction of distant metastasis-free survival (DMFS) of prognostic genes. **A**, **B** In the Kaplan–Meier plots, patients with high-risk scores (above the median value of risk scores) were more likely to develop distant metastasis than those with low-risk scores using training data (**A**), validation data (**B**). **C**–**G** The nomogram model is based on nine prognostic genes for 3-year, 5-year, and 7-year DMFS using training cohorts (blue lines) and validation cohorts (red lines). **E**–**G** The calibration plots of the nomogram for predicting the probability of DMFS at 3- (**E**), 5- (**F**), and 7- (**G**) year

DMFS time was significantly increased in the low-risk group compared with the high-risk group (*p* =  < 0.001; Table 3, Fig. 4A). The risk stratification capability of the risk scores were validated using the independent dataset GSE20685. Similarly, in validation data, risk scores could discriminate between high-risk and low-risk groups (HR 5.48, *p* = 0.003, in Fig. 4B). Next, we entered the risk score and clinical variables of age, lymph-node status, ER, PR, Grade, and PAM50 subtyping in stepwise Cox regression. Her2 was not included due to too much missing data. The risk score and ER status were kept in the final model. The comparison of ROC curves showed that AUC is 0.79 for risk score-ER model, and AUC is 0.77 for risk score model (Fig. 4B). The minor difference indicated the good prediction capability of risk score only. We furtherly evaluated the relationship between risk scores and clinical characteristics (Table 3). Risk scores were significantly associated with DMFS, Grade, ER, PR, and PAM50 subtyping.

**Table 3:** The association of genetic risk score and clinical characteristics using training cohort and univariable Cox regression

	Risk score			*p* value
	Low	High	Total	
	(*n* = 299)	(*n* = 299)	(*N* = 598)	
Distant metastasis				
No	273 (91.3%)	201 (67.2%)	474 (79.3%)	< 0.001***
Yes	26 (8.7%)	98 (32.8%)	124 (20.7%)	
DMFS time				
Mean (SD)	11.2 (6.40)	8.81 (6.27)	10.0 (6.44)	< 0.001***
Median [min, max]	9.81 [0, 26.3]	7.66 [0, 25.9]	8.45 [0, 26.3]	
Age				
Mean (SD)	59.9 (12.5)	56.6 (11.8)	58.3 (12.2)	0.005**
Median [min, max]	60.0 [34.0, 86.0]	56.0 [30.0, 89.0]	58.0 [30.0, 89.0]	
Grading				
Grading I	76 (25.4%)	61 (20.4%)	137 (22.9%)	0.003**
Grading II	185 (61.9%)	162 (54.2%)	347 (58.0%)	
Grading III	38 (12.7%)	76 (25.4%)	114 (19.1%)	
Lymph-node status				
−	237 (79.3%)	230 (76.9%)	467 (78.1%)	0.787
+	62 (20.7%)	69 (23.1%)	131 (21.9%)	
ER				
−	18 (6.0%)	70 (23.4%)	88 (14.7%)	< 0.001***
+	281 (94.0%)	229 (76.6%)	510 (85.3%)	
PR				
−	69 (23.1%)	108 (36.1%)	177 (29.6%)	0.002**
+	230 (76.9%)	191 (63.9%)	421 (70.4%)	
HER2				
−	23 (7.7%)	23 (7.7%)	46 (7.7%)	0.896
+	6 (2.0%)	8 (2.7%)	14 (2.3%)	
PAM50				
Luminal A	8 (2.7%)	57 (19.1%)	65 (10.9%)	< 0.001***
Luminal B	8 (2.7%)	45 (15.1%)	53 (8.9%)	
Her2-enriched	172 (57.5%)	76 (25.4%)	248 (41.5%)	
Basal-like	85 (28.4%)	88 (29.4%)	173 (28.9%)	
Normal-like	26 (8.7%)	33 (11.0%)	59 (9.9%)	

The risk score is categorized into high and low by the median value of risk scores

*DMFS* distant metastasis-free survival.

### Nomogram model construction and visualization

The nomogram model was used to visualize prognostic genes in 3-, 5-, and 7-year DMFS prediction (Fig. 4C, D). The C-index of the nomogram using the training cohort was 0.76 (95% CI 0.72–0.80, *p* < 0.001) and 0.72 (95% CI 0.56–0.88, *p* = 0.017) in the validation cohort. A calibration diagram was also used to verify the prediction ability of risk score for 3-year, 5-year, and 7-year DMFS (Fig. 4E–G). In the calibration plots, the colored solid line was the prediction for DMFS, and the diagonal dotted line was the actual DMFS. The closer the solid line was to the dotted line, the better the prediction ability was. The calibration curves of the 3-year, 5-year, and 7-year DMFS showed good agreements between predicted DMFS and observed DMFS (Fig. 4E–G). These results suggested that our nomogram had good prognostic significance.

### Weighted gene co-expression network analysis

**Fig. 5:**
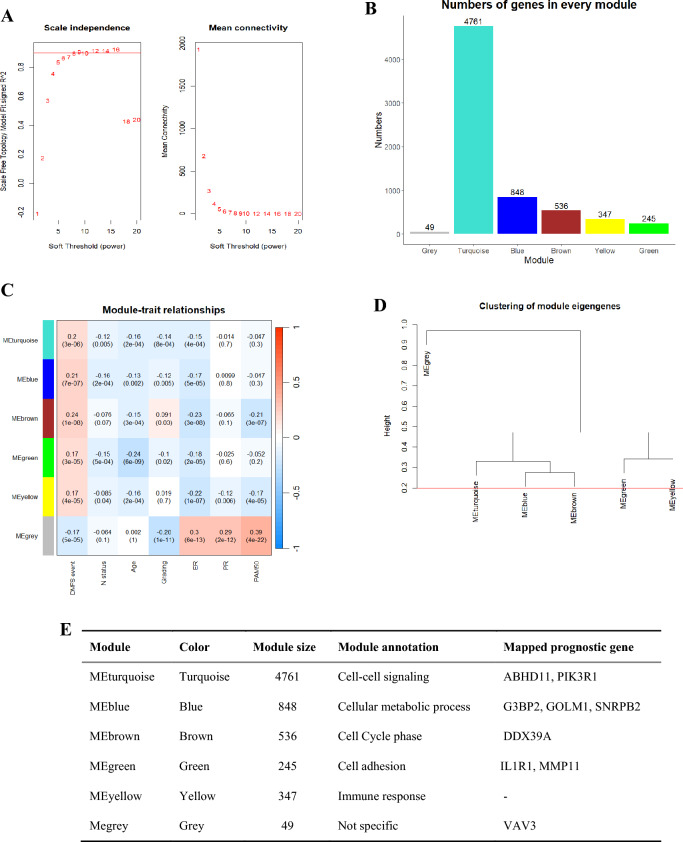
The genetic modules of 6,786 significant genes related to distant metastasis using weighted gene co-expression network analysis. **A** The plot of soft threshold, scale independence and mean connectivity. **B** The number of genes in modules. **C** The heatmap of module-trait relationship. **D** Clustering of module eigengenes. **E** Information of modules

A WGCNA network was constructed using the training cohort. With WGCNA applying scale-free topology criterion, the soft threshold power of *β* was 6 when scale‑free topology model fit *R*^2^ was maximized (0.9), and the mean connectivity for the network was 6. A total of six modules were identified (module size ≥ 100 and cut height ≥ 0.2) in the network (turquoise, blue, brown, yellow, green, and grey; Fig. 5A). The number of genes comprising each module is shown in Fig. 5B.

Except for the grey module, all the modules were positively associated with distant metastasis and negatively associated with lymph-node status, age, grading, ER, PR, and PAM50 subtypes (Fig. 5C). According to results of module annotation, each of the five modules was associated with cell–cell signaling (ABHD11 and PIK3R1), cellular metabolic process (G3BP2, GOLM1, SNRPB2), cell cycle phase (DDX39A), cell adhesion (IL1R1 and MMP11) and immune response. VAV3 was clustered in MEgrey (Fig. 5D).

### Functional analysis

**Fig. 6:**
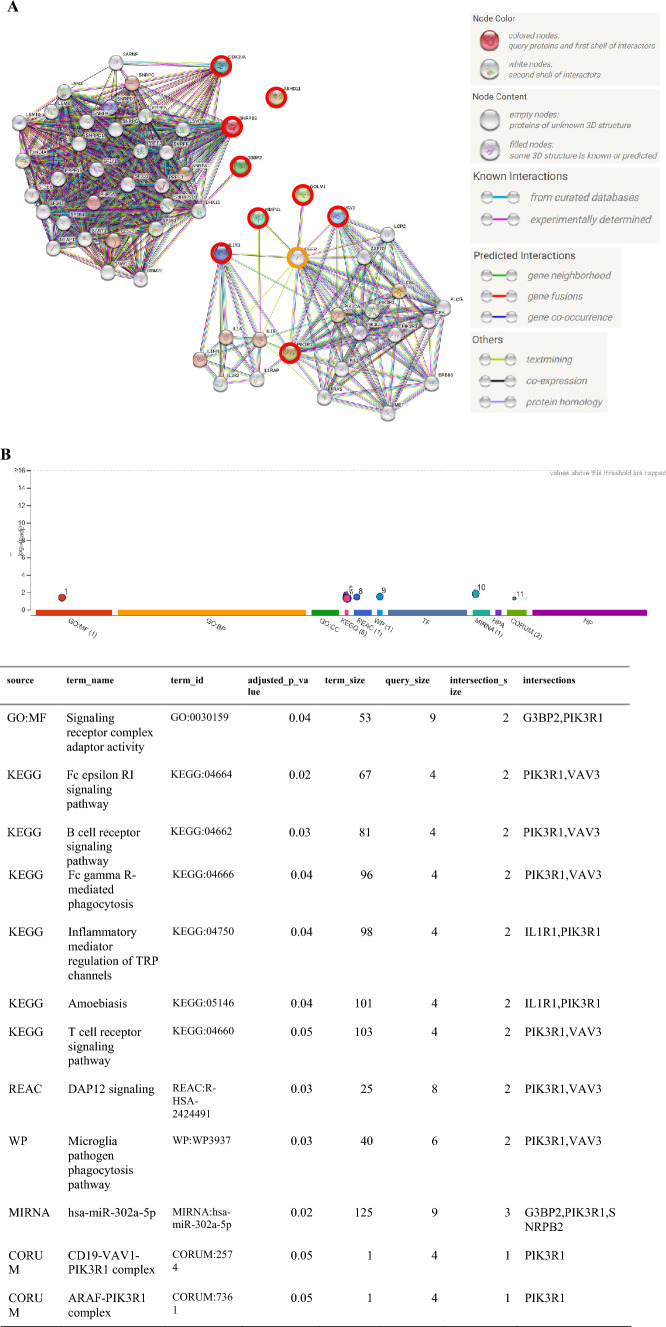
The functional annotation of nine prognostic genes using STRING (**A**) and gProfiler (**B**)

The extended protein–protein (PP) interaction of nine prognostic genes is shown in Fig. 6A. Of note, IL1R1, MMP11, GOLM1, VAV3, and PIK3R1 were connected by the hub gene EGFR. DDX39A, SNRPB2, and G3BP2 were in the same PP interaction network. These interactions conformed to the finding in the clustering result of the heatmap (Fig. 3B). The functional analysis result of gProfiler demonstrated that G3BP2 and PIK3R1 were related to signaling receptor complex adaptor activity. Additionally, PIK3R1 and VAV3 were related to host-defense mechanisms; IL1R1 and PIK3R1 were related to immune functions; G3BP2, PIK3R1, and SNRPB2 were co-regulated by hsa-miR-302a-5p (Fig. 6B).

## Discussion

Traditionally, the size of breast cancer at diagnosis is seen as a key determinant of clinical outcome. However, some aggressive subtypes challenge this notion despite being small (≤ 1 cm) [6]. In certain subtypes, tumor size, lymph-node status, and prognosis may be uncoupled due to a disproportionate number of metastatic cancer cells relative to tumor size [5]. Therefore, understanding the underlying mechanisms of distant metastasis in small-size tumors is an important clinical issue for appropriate treatment decisions. We used machine learning Random Survival Forest and WGCNA techniques to identify nine prognostic genes (ABHD11, DDX39A, G3BP2, GOLM1, IL1R1, MMP11, PIK3R1, SNRPB2, and VAV3) that were predictive of DMFS. Their functions were related to “cell adhesion” (IL1R1 and MMP11), “cell–cell signaling” (ABHD11 and PIK3R1), “cellular metabolic process” (G3BP2, GOLM1 and SNRPB2), “cell cycle phase” (DDX39A), and “not specific” (VAV3) from the results of WGCNA. Patients with higher risk scores had a three-to-fourfold increased risk of developing distant metastasis. When ER status was added, the risk score had good prediction efficacy, with AUCs of 0.75 and 0.79. Furthermore, the risk score reflected clinical characteristics such as lower age, poor Grading, ER-, PR-, and a higher proportion of luminal A/B.

In this study, we employed nomogram models to simplify and visualize prognostic genes in the prediction of DMFS shown in Fig. 4C, D. By obtaining the normalized gene expression of these nine genes, we can determine the individual corresponding scores by referring to the “Points” scales and summing them to derive the “Total Points.” For example, using the nomogram model in Fig. 4D, a hypothetical patient with the following values: DDX39A (12.2 mapping to 100 points), VAV3 (11 mapping to 10 points), GOLM1 (5 mapping to 0 points), MMP11 (11.5 mapping to 20 points), G3BP2 (12.5 mapping to 99 points), PI3KR1 (10.8 mapping to 10 points), ABHD11 (7.5 mapping to 0 points), IL1R1 (8 mapping to 0 points), and SNRPB2 (12.2 mapping to 0 points) would accumulate a total of 239 (100 + 10 + 0 + 20 + 99 + 10 + 0 + 0 + 0) points. These total points correspond to an estimated 3-year survival probability of approximately 73%, a 5-year survival probability of around 59%, and a 7-year survival probability of about 57%.

Previous molecular studies identified genetic markers involved in the prognosis of small breast tumors. For instance, the expression of stromal type IV, an extracellular matrix protein, in small invasive breast cancers has been linked to a higher risk of developing distant metastasis and poorer survival outcomes. It is possible that stromal type IV collagen can promote metastasis formation by supporting cancer cell survival and tumor progression, and high levels of type IV collagen in the metastases appear to be beneficial for metastatic growth [8]. The expression of P-cadherin has been found to be highly predictive of a poor prognosis in small, node-negative breast cancers. P-cadherin has an important role in maintaining the structural integrity of the epithelium [9]. These studies highlighted the dysregulation of the extracellular matrix in the progression of small breast tumors, consistent with our findings on IL1R1 and MMP11, which are involved in “cell adhesion”. In addition, significant correlation of IL1R1 with MMP11 expression was found and involved in breakdown of extracellular matrix, tissue remodeling, and metastasis [10]. Breast tumors infiltrated by MMP-11+ mononuclear inflammatory cells are more likely to metastasize, have high levels of interleukin (IL)-1, IL-5, IL-6, IL-17, interferon (IFN), and NFB, and an increased CD68+/(CD3+CD20+) cell ratio at the invasive front. These factors are implicated in the crosstalk between tumors and their inflammatory microenvironment [11]. MMP11 expression in mononuclear inflammatory cells was associated with shorter relapse-free survival and overall survival [12].

Early in tumorigenesis, IL-1R1 signaling suppresses mammary tumor cell proliferation and inhibits breast cancer outgrowth and pulmonary metastasis. In breast cancer, IL-1-mediated IL-1R1 signaling is tumor-suppressive [13]. Patients treated with anti-estrogen therapy have increased IL1R1 expression, which predicts treatment failure [14]. In addition, inhibition of IL-1 signaling with the anti-IL1β antibody or the IL1R antagonist inhibits bone metastasis in pre-clinical models of breast cancer [15]. Colorectal cancer patients who did not respond to Cetuximab blockage had higher levels of IL1R1 than responsive subjects, and high levels of IL1R1 are predictive of survival [16]**.**

In this study, we identified PIK3R1-IL1R1-MMP11-GOLM1-VAV3-EGFR protein–protein interaction network connected by Epidermal Growth Factor Receptor (EGFR). EGFR and its downstream pathway regulate epithelial–mesenchymal transition, migration, and tumor invasion and that high EGFR expression is an independent predictor of poor prognosis in inflammatory breast cancer**. **Targeting EGFR enhances the chemosensitivity of tumor cells by rewiring apoptotic signaling networks in Triple-Negative Breast Cancer [17]. Therefore, this genetic network may play crucial role in triggering small-size breast tumor metastases.

GOLM1 has been identified as a potential target for cancer therapy, because it is overexpressed in many solid tumors, promotes tumor growth and metastasis, and leads to poor survival [18]. GOLM1 could promote breast cancer cell aggressiveness by regulating matrix metalloproteinase-13 (MMP13) [19]. Knocking down GOLM1 expression further increased the epigallocatechin gallate (a natural migration-inhibiting substance) treatment effect in breast cancer cells [18]. What’s more, GOLM1 also acts as a positive regulator of Programmed Cell Death Ligand 1 (PD-L1) expression via the EGFR/Signal Transducer and Activator Of Transcription 3 (STAT3) signaling pathway in the human hepatocellular carcinoma [20].

VAV3, a GEF for Rho family GTPases, belongs to the VAV protein family [21]. It is a downstream signal transducer of EGFR/HER2 and could bind to several partners, including PI3K, modulates cell morphology, and induces cell transformation [22]. High nuclear VAV3 expression in tumor cells was associated with poorer endocrine therapy response [23]. It complexes with ERα and together enhance ERα-mediated signaling axis, participating in breast cancer development and/or progression [24]. The depletion of VAV3 reduced the viability of cell models of acquired endocrine therapy resistance [23].

In this study, we unrevealed that G3BP2, PIK3R1, and SNRPB2 were co-regulated by hsa-miR-302a-5p. The MiR-302 family exerts antitumor effects in several cancers [25]. MiR-302a, -b, -c, and -d were found to cooperatively inhibit BCRP expression to increase the drug sensitivity of breast cancer cells [26]. Dysregulation of the phosphoinositide 3-kinase (PI3K) pathway contributes to the development and progression of tumors. PIK3R1 underexpression is an independent prognostic marker in breast cancers [27]. Silencing PIK3R1 enhanced the sensitivity of breast cancer cell lines to rapamycin [28], implicating a negative role of PIK3R1 in PI3K pathway activation. Both PIK3R1 and EGFR were involved in anti-cancer drug effects. Schisandrin A (SchA), a good anti-cancer drug, significantly down-regulated EGFR, PIK3R1, and MMP9 but up-regulated cleaved-caspase 3, thus inhibiting the migration and promoting the apoptosis of MDA-MB-231 cells [29].

G3BP2 (G3BP Stress Granule Assembly Factor 2) regulates breast tumor initiation by stabilizing squamous cell carcinoma antigen recognized by T cells 3 (SART3) mRNA. The loss of G3BP2 inhibits breast tumor initiation, possibly lead to improved cancer treatments [30]. Cell-cycle checkpoint regulator MK2 or G3BP2 inactivation sensitizes cisplatin-resistant TNBC cell lines to cisplatin [31]. Suppression of G3BP2 inhibits the immune checkpoint molecule PD‐L1 due to mRNA degradation [32].

SNRPB2 (Small Nuclear Ribonucleoprotein Polypeptide B2) is the encoding gene of protein U2 small nuclear ribonucleoprotein B, one component of spliceosome. While primarily studied in hepatocellular carcinoma [33], SNRPB2 is a novel gene of interest in breast cancer. Many oncogenic insults deregulate RNA splicing, often leading to hypersensitivity of tumors to spliceosome-targeted therapies (STTs). Mis-spliced RNA is a molecular trigger for tumor killing through viral mimicry. STTs cause widespread cytoplasmic accumulation of mis-spliced mRNAs, many forming double-stranded structures in MYC-driven triple-negative breast cancer [34].

DDX39 encodes protein DExD-Box Helicase 39A that unwinds double-stranded RNA in an ATP-dependent manner. It is involved in transcription, splicing, ribosome biogenesis, RNA export, RNA editing, RNA decay, translation, and the protection and maintenance of telomeres. Increased DDX39 mRNA expression was associated with poor outcomes in ER-positive breast cancers. Inhibiting DDX39 could enhance the sensitivity of MCF-7 to doxorubicin [35]. In hepatocellular carcinoma (HCC), DDX39 knockdown inhibited HCC cell migration, invasion, growth, and metastasis by activating the Wnt/β-catenin pathway [36].

ABHD11 (Abhydrolase Domain Containing 11) is a protein-coding gene. ABHD11 antisense RNA 1 (ABHD11-AS1) is highly expressed in many cancers. Several studies have highlighted the clinical importance of ABHD11-AS1 in cancer prognosis, diagnosis, stage prediction, and treatment response. The ABHD11-AS1 has been shown to cause cancer by sponging various microRNAs (miRNAs), altering signaling pathways such as PI3K/Akt, epigenetic mechanisms, and N6-methyladenosine (m6A) RNA modification [37]. ABHD11 and Esterase D could predict the development of distant metastases and the presence of aggressive lung adenocarcinomas [38].

In the present study, although prognostic genes were validated in the independent dataset, further testing on clinical data is warranted. HER2 status, Ki67, and lymphovascular invasion were not included for analysis due to too much missing data. It should be included in the future work. In addition, the current mRNA expression data require experimental studies to validate the findings and elucidate the mechanisms.

## Conclusions

In sum, we utilized machine learning and WGCNA of large-scale data from the GEO database to identify prognostic gene set of ABHD11, DDX39A, G3BP2, GOLM1, IL1R1, MMP11, PIK3R1, SNRPB2, and VAV3 involved in the distant metastasis for BC patients with small tumor size. They are reportedly linked to metastasis and treatment resistance. When compared to a low genetic risk score, a high genetic risk score comprising nine genes predicted poor DMFS. To note, the protein–protein interaction network of PIK3R1, IL1R1, MMP11, GOLM1, and VAV3 altogether connected by EGFR merits further work to understand the mechanism and develop an ideal treatment strategy for invasive small tumor-size breast cancers.

## Supplementary Information

Below is the link to the electronic supplementary material.

Supplementary file1 (DOCX 18 KB)

## Data Availability

All data can be downloaded from Gene Expression Omnibus (https://www.ncbi.nlm.nih.gov/geo/).

## Abbreviations

BC: Breast cancer
GEO: Gene Expression Omnibus
DMFS: Distant metastasis-free survival
DM: Distant metastasis
HR: Hazard ratio
WGCNA: Weighted gene co-expression network analysis
CCLE: Cancer cell line encyclopedia
Depmap: Dependency map
