# Supplementary material for: Hub metastatic gene signature and risk score of breast cancer patients with small tumor sizes using WGCNA
Source: Breast Cancer. 2024 Aug 27;31(6):1114–29. doi: 10.1007/s12282-024-01627-w (PMC11489208; doi:10.1007/s12282-024-01627-w)
Supplement: Supplementary file 1 — Supplementary file1 (DOCX 18 KB) [file 12282_2024_1627_MOESM1_ESM.docx]

# Supplementary

## Table S1 GEO datasets from NCBI

|  | Dataset | N | DMFS time | DMFS status | Tumor size | Lymph node | Grading | ER | PR | HER2 | platform |
| --- | --- | --- | --- | --- | --- | --- | --- | --- | --- | --- | --- |
| Training set | GSE25066 | 508 | v | v | v | v | v | v | v | v | GPL96 |
|  | GSE45255 | 139 | v | v | v | v | v | v | v | v | GPL96 |
|  | GSE11121 | 200 | v | v | v | v | v |  |  |  | GPL96 |
|  | GSE6532 | 327 | v | v | v | v | v | v | v |  | GPL96 |
|  | GSE158309 | 461 | v | v | v | v | v |  |  |  | GPL96 |
|  | GSE6532 | 87 | v | v | v | v |  | v |  |  | GPL570 |
|  | GSE16446 | 120 | v | v | v | v | v |  |  |  | GPL570 |
|  | GSE9195 | 77 | v | v | v | v | v | v | v |  | GPL570 |
|  | GSE58984 | 94 | v | v | v | v |  | v | v |  | GPL570 |
| Validation set | GSE20685 | 327 | v | v | v |  |  |  |  |  | GPL570 |

## 
